# Pore-Forming Proteins as Mediators of Novel Epigenetic Mechanism of Epilepsy

**DOI:** 10.3389/fneur.2017.00003

**Published:** 2017-01-18

**Authors:** Andrei Surguchov, Irina Surgucheva, Mukut Sharma, Ram Sharma, Vikas Singh

**Affiliations:** ^1^Department of Neurology, Kansas University Medical Center, Kansas City, KS, USA; ^2^Kansas City Veterans Administration Medical Center, Kansas City, MO, USA; ^3^Midwest Biomedical Research Foundation, Kansas City, MO, USA

**Keywords:** epilepsy, ion channels, α-synuclein, stefin B, epigenetic mechanisms, posttranslational modifications, microRNA, Parkinson’s disease

## Abstract

Epilepsy is a disorder of the brain characterized by an enduring predisposition to generate epileptic seizures. In the last two decades, numerous gene defects underlying different forms of epilepsy have been identified with most of these genes encoding ion channel proteins. Despite these developments, the etiology of majority of non-familial epilepsies has no known associated genetic mutations and cannot be explained by defects in identified ion channels alone. We hypothesize that *de novo* formation of ion channels by naturally unfolded proteins (NUPs) increases neuronal excitability. Altered ionic homeostasis may initiate/contribute to cellular cascades related to epileptogenesis in susceptible individuals. Here, we consider two small proteins, namely, α-synuclein and stefin B, as prototypical candidates to illustrate the underlying mechanism(s). Previous work points to an association between epilepsy and α-synuclein or stefin B, but the mechanism(s) underlying such association remains elusive. We review the evidence to link the structure–function of these proteins with disease processes. Epigenetic mechanisms unrelated to altered DNA sequence(s) that may affect epileptogenesis include transcriptional or posttranscriptional regulation. Such epigenetic mechanisms or their combination(s) enhance the levels of these proteins and as a result the ability to form annular structures, which upon incorporation into membrane form novel ion channels and disturb intracellular ion homeostasis. Alternative epigenetic mechanisms may change amyloidogenic proteins by posttranslational modifications, thereby increasing their propensity to form channels. Further research elucidating the details about the formation of ion channels through these mechanisms and their role in epileptogenesis may define new molecular targets and guide the development of new drug targets.

## Introduction

Epilepsy is a group of neurological diseases characterized by recurrent episodes of convulsive seizures, sensory disturbances, abnormal behavior, loss of consciousness, or a combination of these signs and/or symptoms. As defined by International League Against Epilepsy, epilepsy is a disorder of the brain characterized by an enduring predisposition to generate epileptic seizures that are more than 24 h apart (1). Although epilepsy can manifest itself in a number of different ways, it shares the common feature of persistently increased neuronal excitability and spontaneous seizure generation.

Epilepsy is a widespread disease, being the fourth most common neurological disorder in the United States after migraine, stroke, and Alzheimer’s disease. Approximately 150,000 new cases are diagnosed in the United States annually, and 1 in 26 individuals will develop epilepsy at some point in their lifetime. The total indirect and direct cost of epilepsy in the United States is estimated to be $15.5 billion per year (2).

Despite recent advancements in epilepsy treatment, currently available interventions and antiepileptic drugs (AEDs) are not effective in over 30% of patients (3). The current state of known mechanisms of epilepsy and the range of available treatments suggest additional pathways and mechanisms responsible for currently intractable cases of epilepsy. This underscores the importance of more research needed to better understand the underlying disease mechanisms and identify novel drug targets or treatment strategies (4). We present available evidence for the potential role of epigenetic mechanisms and certain naturally unfolded proteins (NUPs) that may create pores in cell membranes and contribute to dysregulated ion flows that characterize electric discharges.

## Mechanism of Idiopathic Epilepsy is Not Clear

Genetic mutations play an important etiological role in the development of epilepsy in hereditary cases of this disease (5–10). However, the mechanism of seizures in a significant proportion of patients remains unexplained. Such individuals include those with traumatic brain injury (TBI), intracranial infection, brain tumor, stroke, vascular disturbances, intoxication, or chemical imbalance. Additionally, there exists a large cohort of patients who may not be accounted for under these categories of associated conditions. Development of such idiopathic epilepsies represents a spectrum of changes from initial injury to established disease state. These changes include molecules to pathways and single cell structure to cellular network organization.

Epileptogenesis summarizes interactive changes in cellular processes that culminate in the development of a epilepsy. Epileptogenesis spans over a period without overt manifestation of symptoms, while subcellular structural and functional alterations may occur. These changes include neuronal death, axonal growth and sprouting, altered inter neuron connections, and reorganization of neuronal networks. Temporal connections among these changes or their relationships with the onset of neuronal hyperexcitability and spontaneous seizures are not well understood. Cellular events during epileptogenesis may include epigenetic mechanisms that modify processing of RNA and/or proteins leading to non-familial epilepsy.

## Defects in Ionic Channels Cause Seizures

Common to all types of epilepsy is an uncontrolled electrical discharge from neurons. Therefore, ion channels are recognized as the key target in understanding the mechanism and treatment of epilepsy. Ion channels constitute a unique group of cellular proteins, which are highly significant in excitable cells due to their role in generation and regulation of electrical discharges. Ion channels are formed by multimeric arrangements of ionophoric membrane proteins. These proteins organize to provide a passageway (channel) across the membrane connecting its outer and inner sides. Such channels permit a controlled flow of ions across the two sides of membrane regulating the resting potential, action potential, and other electrical properties of the membrane while modulating cellular responses to the microenvironment. Ion channels also contribute to regulated cellular responses to changes in cellular environment and maintain ion homeostasis.

Ion channels constitute the most favored class of targets for currently available treatments. Most AEDs have been designed to target ion channels and transporters and one that affects synaptic vesicle protein Sv2a with a goal to favor inhibition of over excitation and thereby decrease or prevent seizure activity/frequency (11–13). These include AEDs targeting voltage-gated sodium channels, voltage-gated calcium channels, voltage-gated potassium channels, the GABA system and receptor agonists, glutamate receptor antagonists, and other inhibitory neurotransmitters. Additionally, currently used AEDs reduce neuronal hyperexcitability and thereby suppress epileptic seizures.

Despite these developments, available AEDs have had only limited success. Current drugs, more than 20, are employed solely to suppress seizures. Seizures can be effectively controlled in ~70% of patients, whereas 30% remain refractory to drug treatment pointing to alternative mechanisms and significant unmet need for effective intervention. Second, the existing armamentarium does not affect the comorbidities of epilepsy and does not prevent the development and progression of epileptogenesis (14–16). By and large, the entire process of epileptogenesis offers a number of targets but remains underappreciated.

Altered ion channel function is used as a leading reason of the etiology of seizures and epilepsy. However, lack of explanation for disease manifestation in the absence of gene mutations represents gap in our understanding of the mechanism(s) of the disease. Thus, altered ion homeostasis while ion channels are not genetically altered remains an unanswered question. With these problems in view, a better understanding of the events of epileptogenesis may lead to prevention of seizures, early diagnoses, and treatment of epilepsy in those patients who do not benefit from current treatments.

## Epigenetic Mechanisms Control Gene Function without DNA Mutation

Recent developments show that DNA may undergo multiple enzymatic modifications, which are used in epigenetic regulation of gene expression. Epigenetics is the study of alterations in the function of genes that do not involve changes in the DNA sequence itself, but rather its expression. Epigenetic mechanisms involve the transfer of information arising from short-lived cellular signals and changes in neuronal activity into lasting effects on gene expression.

Rapid exponential advances in high-throughput DNA sequencing technologies convincingly demonstrate that a considerable number of epileptic patients do not carry mutations in genes encoding ion channels or related genes, thereby implying alternative mechanisms involved. Therefore, it is important to learn what other molecular mechanisms, not associated with alterations in genome sequence, may contribute to the development of epilepsy.

Importantly, from the viewpoint of treatment, epigenetic changes are potentially reversible and therefore may form the basis for the future development of efficient therapies. Major epigenetic mechanisms include DNA methylation, histone modification, and microRNA-driven posttranscriptional regulation of gene expression. Methylation-dependent gene silencing is currently one of the most thoroughly investigated mechanisms for regulation of gene expression (17–21).

## Epilepsy such as TBI-Induced Epilepsy may be Caused by Epigenetic Mechanisms

Epigenetics may explain the process of epileptogenesis such as posttraumatic epilepsy (PTE) in TBI. Although the study of epigenetic mechanisms of PTE is an emerging area of investigation, the results of several studies point to the importance of this approach in understanding the consequences of TBI in general and PTE in particular. For example, Gao et al. (22) demonstrated that TBI may cause epigenetic changes in an animal model of TBI. Specifically, alterations in hippocampal CA3 histone H3 acetylation and methylation occur hours to days following injury. In another study, global hypomethylation was observed after TBI in a subpopulation of reactive microglia/macrophages (23). An important, but not completely understood clue to understanding of TBI-induced epigenetic alterations is the finding that TBI causes relocation of DNA-methyltransferase 1—enzyme catalyzing sequence-specific DNA methylation (24). However, a detailed mechanism underlying this translocation and its connection with methylation pattern changes require further studies.

## Idiopathic Forms of Epilepsy may be Associated with the Formation of New ION Channels by NUPs

Posttraumatic epilepsy is just one example of the development of epilepsy without an underlying, inherited mutation(s). Below we discuss another example based on the formation *de novo* ion channels from amyloidogenic proteins, which may be responsible for non-familial cases of epilepsy.

**Figure 1:**
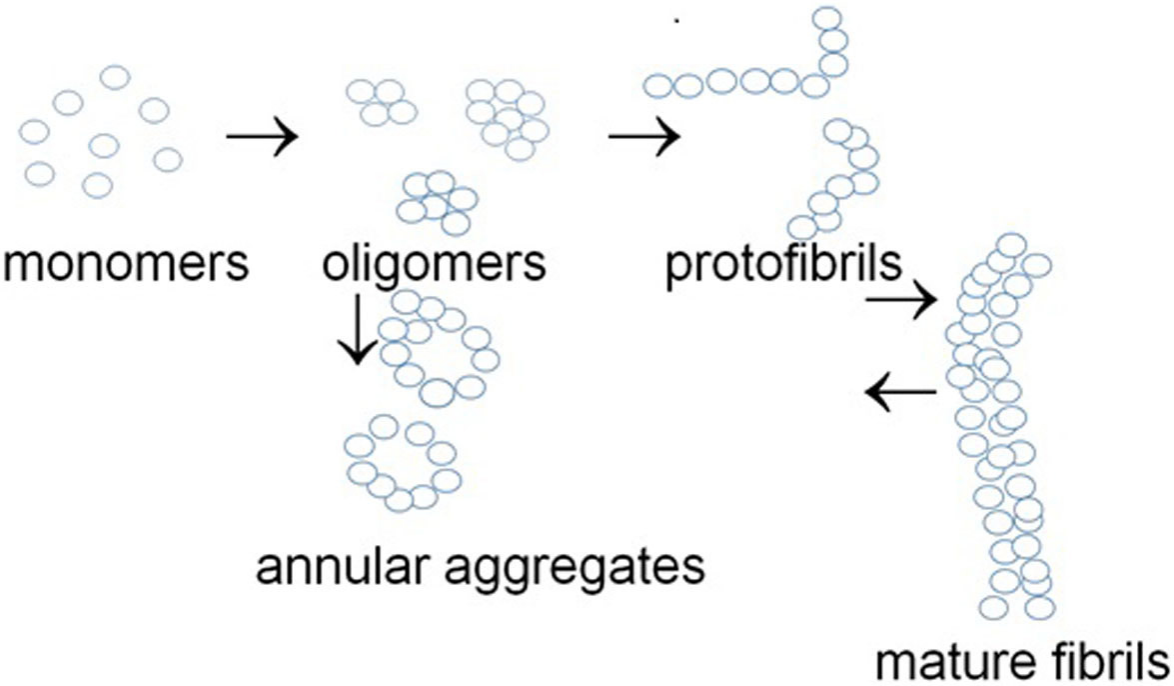
**Examples of various aggregated states of naturally unfolded proteins**.

In addition to the heterogeneous family of authentic ion channels, a group of naturally unfolded intrinsically unstructured proteins can form novel ion channels as a result of their conformational transitions. This group consists of the so-called NUPs, which can aggregate to form oligomers and amyloid structures. These proteins may accumulate as large amyloid deposits and inclusions in Alzheimer’s disease (amyloid beta, Aβ) (25–27), Parkinson’s disease (α-synuclein), and prion disease (prion protein, PrP) (28–33). Alternatively, NUPs can form annular protofibrils that can act as ion channels increasing membrane conductance (34, 35) (Figure 1).

The formation of ion channels from NUPs is a well-known phenomenon (35–37). However, the role of such channels generated under *de novo* conditions has not been investigated to explore the development of epilepsy.

Ion channel-like membrane pores may be ion-specific or non-selective with regard to ions. Amyloidogenic proteins generally produce rather large pores (3–10 nm in diameter), which are relatively non-selective for ion traffic (38, 39). However, well-defined sizes and morphologies have been found for some amyloid pores such as those generated from α-synuclein (34).

## α-Synuclein and Stefin B are Candidate Pore-Forming Proteins

We present below several observations showing that proteins forming annular protofibrils and thus affecting membrane conductance are associated with epilepsy. We will discuss here two NUPs that can form membrane pores or channels, namely, α-synuclein and stefin B. These proteins have several similar properties, which may determine their ability to form new ionic channels and therefore a role in epilepsy:

(a) These are small size proteins that can readily interact with membranes to form oligomers, annular structures, and amyloid fibrils (40, 41, 42).
(b) Their properties are regulated by posttranslational modifications (43, 44).
(c) A lack of cysteine prevents formation of disulfide bonds in both proteins (45–47).
(d) Expression of α-synuclein and stefin B is regulated by epigenetic mechanisms, such as methylation/demethylation of regulatory regions in their genes (48, 49).
(e) Both proteins lack export signal sequences; however, they are detectable in both intracellular and extracellular spaces (45, 47).

**Figure 2:**
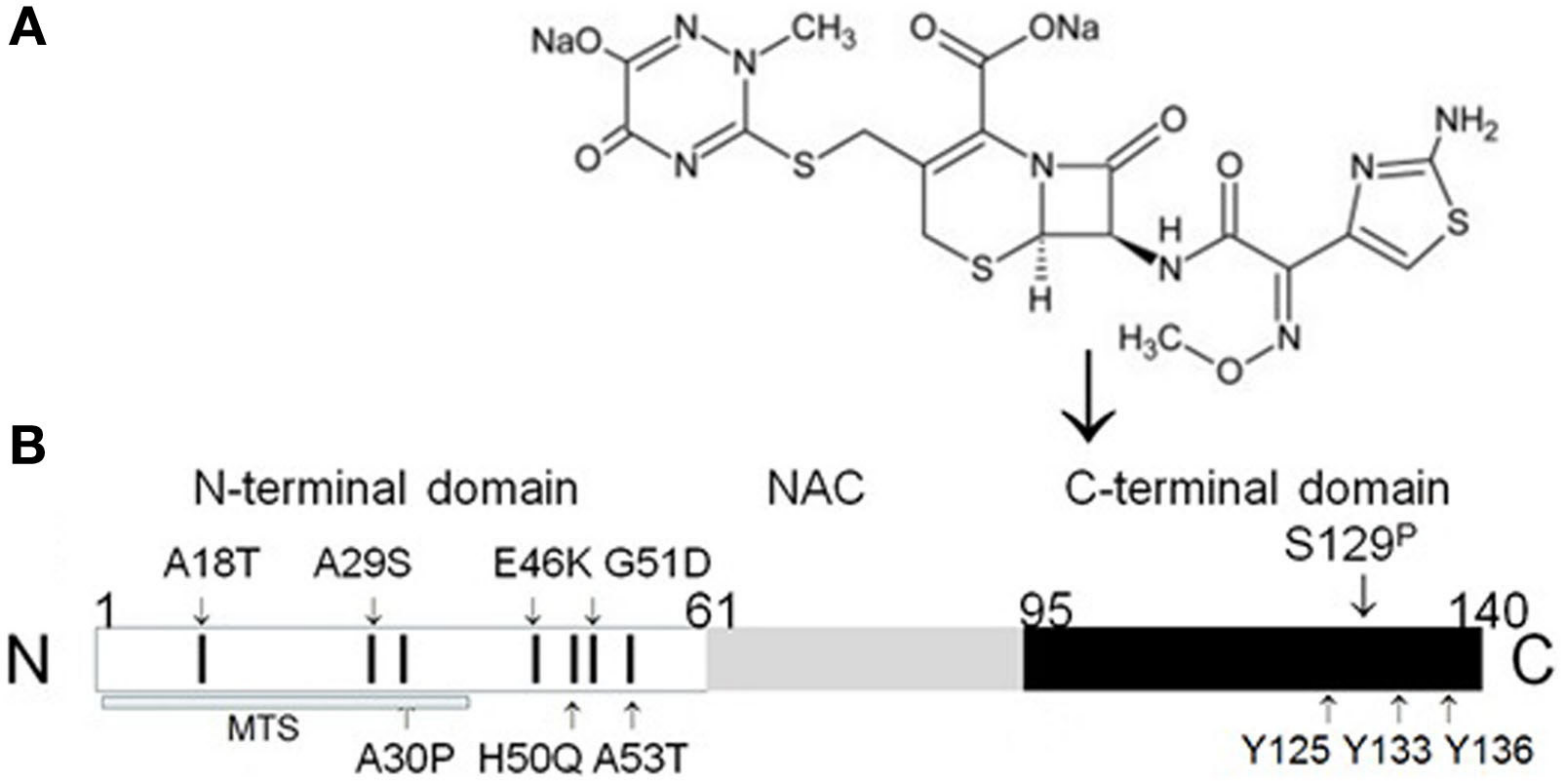
**Binding of β-lactam antibiotic ceftriaxone to α-synuclein C-terminus**. **(A)** Chemical structure of ceftriaxone. **(B)** Schematic organization of human α-synuclein with localization of known missense mutations indicated by small arrows. N-terminal domain contains all missense mutations, predisposing to α-synuclein aggregation. These mutations include three well-characterized and carefully studied missense mutations: A53T (51), A30P (52), and E46K (53). The toxicity of these mutant forms of α-synuclein may be due to their enhanced aggregation into oligomers and amyloid fibrils. Two recently identified mutations, which are not yet well investigated, H50Q (54) and G51D (55), may have an important effect on α-synuclein properties, since they add new potential phosphorylation sites to the first N-terminal helix (56). Phosphorylation of Ser129 enhances aggregation of α-synuclein (57). Y125, Y133, and Y136—tyrosine residues, which are a part of ceftriaxone-binding site (58). MTS, mitochondria-targeting sequence (42). Modified from Ghanizadeh and Berk (59).

Conversely, there is a difference between α-synuclein and stefin B with regard to their association with epilepsy. Several missense mutations in stefin B are linked to epilepsy (50), whereas rare missense mutations in α-synuclein gene (Figure 2B) are associated with Parkinson’s disease (51–53).

No data about association of mutations in α-synuclein gene with epilepsy are currently available.

Mechanisms that link Parkinson’s disease with epilepsy require further exploration but are beyond the scope of this review.

### α-Synuclein

α-Synuclein is a major structural component of intracellular protein inclusions or Lewy bodies that constitute a pathological hallmark of Parkinson’s disease. Furthermore, α-synuclein is implicated in multiple system atrophy, dementia with Lewy bodies, and other diseases (37, 60). However, the exact mechanism of α-synuclein toxicity and its role in healthy cells and in diseases remain elusive. While a direct link between mutations in α-synuclein and epilepsy has not been described so far, several findings point to an association of α-synuclein with this disease. These include the following:

(a) Patients with α-synuclein gene multiplication have seizures. Although a rare event, multiplication of α-synuclein gene resulting in its abundance in the central and peripheral nervous system generates different disease phenotypes, including seizures and myoclonus (61).
(b) α-Synuclein concentration is elevated in the CSF and serum of epileptic patients (62), as well as in a mouse model of epilepsy (63) (see details in Section “α-Synuclein Concentration Is Changed in Body Fluids of Epileptic Patients”).
(c) β-Lactam antibiotics, such as ceftriaxone, known to reduce frequency of seizures bind to α-synuclein and inhibit its aggregation (58).

We outline the physicochemical characteristics of α-synuclein, which render this molecule capable of forming new ion channels.

#### Structure and Functions of α-Synuclein

α-Synuclein is an NUP of the synuclein family, which consists of three members, namely, α-, β-, and γ-synuclein (42, 47). α-Synuclein is a small protein (140 amino acids, 14 kDa) predominantly expressed in CNS neurons. α-Synuclein amino acid sequence can be subdivided into three domains: N-terminal, amphipathic repeat region (residues 1–60), the so-called non-amyloid beta component domain (NAC-region, residues 61–95), and C-terminal acidic domain (residues 96–140) (Figure 2).

In physiological solutions, α-synuclein is characterized by a lack of rigid, well-defined structure and exists predominantly in unfolded or intrinsically disordered form (29, 37, 60). However, α-synuclein possesses high conformational plasticity and is able to adopt a series of different conformations depending on the environmental conditions and binding partners (64). Importantly, formation of oligomers and aggregates is concentration dependent, increases at elevated α-synuclein expression, underlies a toxic gain of function, and may lead to the development of pathology (65–67).

#### α-Synuclein Forms Annular Protofibrils Increasing Membrane Permeabilization

Lashuel et al. (34) described the ability of α-synuclein to form annular protofibrils, which might incorporate into membrane, form pores, and disturb membrane permeabilization leading to cell dysfunction and even cell death. This pathogenic–protofibril hypothesis was supported by biophysical studies of not only α-synuclein but also other amyloidogenic proteins, e.g., Aβ suggesting a putative common mechanism in Parkinson’s and Alzheimer’s diseases (34).

Protofibrillar α-synuclein is able to induce vesicle permeabilization (37), while helical α-synuclein forms highly conductive ion channels (35). Ding et al. (68) described production of annular α-synuclein protofibrils when spherical protofibrils were incubated in solution or bound to brain-derived membranes.

#### α-Synuclein Concentration Is Changed in Body Fluids of Epileptic Patients

Increased α-synuclein concentration in serum, CSF, and specific brain regions has been described in epileptic patients as well as in an animal model of epilepsy. Li et al. (63) used the pilocarpine mouse model of temporal lobe epilepsy to screen the proteome and found a statistically significant increase in α-synuclein expression in temporal lobe epilepsy. In another study, Rong et al. (62) found elevated concentration of total α-synuclein in the CSF and serum of the epilepsy patients, as compared to the control. The authors concluded that α-synuclein content in the serum and CSF may facilitate the identification of intractable epilepsy and serve as a valuable prognostic marker in the clinical assessment. Yang et al. (69) described aberrant expression of α-synuclein in hippocampus from patients with mesial temporal lobe epilepsy. Thus, α-synuclein is increased in body fluids of epileptic patients and can be used as an essential biological marker in epilepsy.

#### β-Lactam Antibiotics with Antiseizure Activity Inhibit α-Synuclein Aggregation

β-Lactam antibiotics, e.g., ceftriaxone or cefixime, possess neuroprotective effect, exhibit excellent blood–brain barrier penetration, and suppress posttraumatic seizures. Ceftriaxone treatment ameliorates both seizure frequency and duration (70). Importantly, these antiseizure substances bind to α-synuclein and affect its aggregation (58).

#### Epigenetic Mechanisms Affecting α-Synuclein Ability to Form Ion Channels

**Figure 3:**
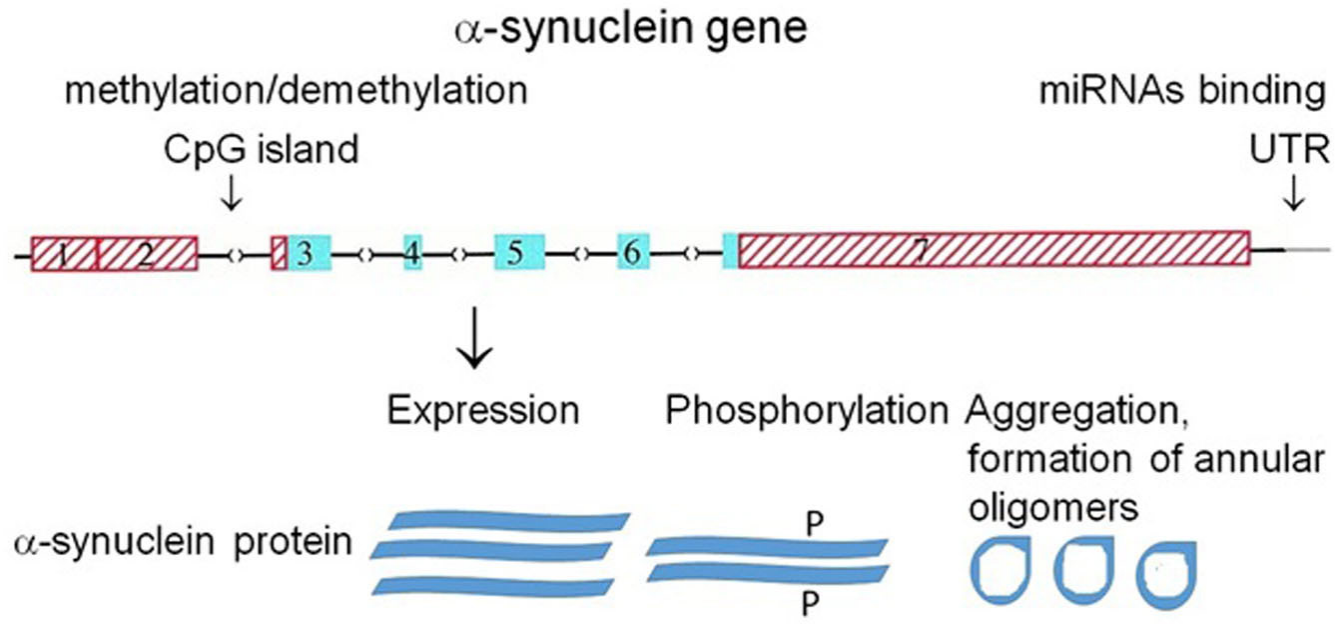
**Putative mechanisms affecting α-synuclein ability to form ion channels**. Organization of human α-synuclein genes (71). Blue (coding) and red (untranslated) exons are shown in boxes; introns are shown as interrupted horizontal lines. Epigenetic mechanism: methylation of CpG islands and microRNA binding to 3′-UTR region of the gene regulate α-synuclein expression on transcriptional and posttranscriptional level, respectively. Phosphorylation (P) of α-synuclein regulates its aggregation.

Several mechanisms may affect α-synuclein ability to form ion channels (Figure 3).

α-Synuclein aggregation is a concentration-dependent process and therefore may be affected by the level of its expression. A number of mechanisms control the level of α-synuclein expression, including transcriptional regulation by methylation/demethylation of regulatory regions in α-synuclein gene (48, 49) (*mechanism #1*). Another mechanism controlling α-synuclein expression is posttranscriptional regulation by microRNAs (43) (*mechanism #2*). Finally, α-synuclein posttranslational modification, e.g., phosphorylation predisposes it to conformational changes and aggregation (44, 57) (*mechanism #3*) and may affect its pore formation capacity. In addition to its ability to form annular and ring-like structures (Figure 1) functioning as ion channels, α-synuclein also interacts with intrinsic ion channels that build from other proteins and changes their properties (72). This feature of α-synuclein may also change conductance through alteration of membrane permeability. Several mechanisms may affect the ability of α-synuclein to form ion channels (Figure 3).

### Stefin B

#### Structure and Function of Stefin B

Human stefin B is a member of cystatin superfamily consisting of single-chain protein, which is composed of 98 amino acid residues with a molecular mass of 11 kDa (45). Stefin B is a neutral protein able to form a dimer stabilized by non-covalent forces. Similar to other members of the cystatin superfamily, stefin B is reversible and competitive inhibitor of cysteine proteases, particularly cathepsin L and cathepsin S. Stefin B is widely distributed among different cell types and tissues. Although it lacks an export signal sequence and is generally thought to function intracellularly, it has also been found in extracellular fluid (45). Stefin B plays a role in protecting cytosolic and cytoskeleton proteins against the cysteine proteases. Besides protease inactivation, stefin B binds other proteins forming a multiprotein complex, which might contribute to the disease in patients with progressive myoclonic epilepsies.

Eight mutations in the stefin B gene have been reported to associate with progressive myoclonic epilepsy of Unverricht–Lundborg type (EPM1). Most of the disease alleles harbor an unstable expansion of at least 30 copies of a normally polymorphic 12-nucleotide, dodecamer repeat located in the promoter region of the stefin B gene. Three reported EPM1 mutations affect splice sites, two result in amino acid changes, and two predict truncated proteins due to a nonsense or frameshift mutation (45).

#### Stefin B Forms Annular Protofibrils Increasing Membrane Permeabilization

Although the function of stefin B in the pathogenesis of epilepsy is not completely understood, experiments *in vitro* show that two missense mutants G50E and Q71P aggregate to a much greater extent than the wild-type (WT) protein (50).

Calcein release results and electrophysiological measurements demonstrate that both WT stefin B and the Y31, but not G4R mutant isoform, are able to form pores in planar lipid bilayers, thereby increasing membrane permeabilization (41). Importantly, there is a clear distinction between oligomer size and toxicity. In the case of WT stefin B, the monomers, dimers, and tetramers of WT stefin B are not toxic, while higher species starting with hexamers and dodecamers acquire toxicity (73).

#### Mechanisms Affecting the Formation of Ion Channels by Stefin B

Mechanisms affecting stefin B expression and regulating its ability to form ion channels are similar to those described for α-synuclein. Stefin B epigenetic mechanisms regulating its expression through DNA methylation are investigated in several diseases, including breast, pancreatic, brain, and lung cancer (46).

Patients with progressive epilepsy of the Unverricht–Lundborg type are characterized by myoclonic seizures and ataxia. The majority of such patients carry repeat expansions of a dodecamer with high GC content in the promoter region of the stefin B gene (74). Although the regulatory role of these dodecamer has not been investigated in detail, these GC stretches are the site of methylation/demethylation regulating the expression of the downstream gene. These findings suggest that DNA methylation-dependent epigenetic mechanisms may play a central role in the regulation of stefin B expression.

The knowledge of epigenetic mechanisms implicated in the development of epilepsy provides a conceptual and mechanistic framework for the future development of epigenetic therapies tailored to prevent epilepsy (antiepileptogenic) or its progression (disease modifying).

## Discussion and Conclusion

Numerous causative mutations have been detected and characterized in familial cases of epilepsy, and the details about genetic mechanisms underlying this disease are accumulating. In contrast, the underlying mechanisms in a significant proportion of epilepsy patients remain unknown, thereby hampering treatment and drug development. We propose novel biochemical and epigenetic mechanisms, which provide alternative explanation for the development of idiopathic epilepsy. We consider here that certain NUPs may form new channels as a result of epigenetic modification(s) on transcriptional (methylation/demethylation of regulatory regions), posttranscriptional levels (microRNA-driven mechanism), or due to posttranslational modifications (phosphorylation, glycosylation of protein, etc.). We provide evidence that NUPs can bind to plasma membrane and form new ion channels that can alter intracellular ion homeostasis.

A role of NUPs in the formation of new channels, which may disrupt membrane conductance, disturb ion homeostasis, and ultimately lead to seizures, is based on several experimental findings:

(a) α-Synuclein and other intrinsically unstructured proteins form annular protofibrils or pores, which incorporate into membrane and act as ion channels increasing membrane permeabilization (34, 35, 37).
(b) Patients with α-synuclein genetic defects have seizures (61).
(c) β-Lactam antibiotics, such as ceftriaxone, known to reduce frequency of seizures bind to α-synuclein and inhibit its aggregation (58).
(d) α-Synuclein concentration is elevated in the CSF and serum of epileptic patients (62) and in a mouse model of epilepsy (63).

Each of these findings individually may be considered only as indirect evidence. However, considering the molecular nature of α-synuclein in the light of an increase in the frequency of seizures in parallel with its levels provides a strong argument in support of a potential role of α-synuclein in epilepsy. Long duration of epileptogenesis provides sufficient time for manifestation of several possible epigenetic mechanisms, which may trigger the formation of novel ion channels. Alternatively, formation of novel ion channels and changes in membrane permeabilization may lead to epigenetic changes.

In fact, the proposed mechanism for ion channel formation may be widely distributed in the cell than currently imagined. In addition to plasma membrane, formation of ion channels may also be evident in intracellular organelles, e.g., membranes of mitochondria and lysosomes. Therefore, many details of epigenetic mechanisms remain to be determined in order to better understand the causes of sporadic epilepsy and their specific treatments.

Several alternative hypotheses have been proposed previously to explain the development of non-familial forms of epilepsy due to epigenetic mechanisms. Among them is an increased activity of DNA methylating enzymes leading to hypermethylation of DNA (75–79). Another proposed mechanism implicates histone modifications that involve the addition or removal of methyl or acetyl groups during epileptogenesis (21). Recently, Boison (16) proposed that adenosine and glycine may regulate epigenetic mechanisms of epileptogenesis. Other putative mechanisms of epileptogenesis are discussed in recent publications (80–83). Our hypothesis is different from those mentioned here. We believe that while acquired epilepsy is associated with ion channels, its etiology and pathophysiology are likely determined by the *de novo* formation of channels from NUPs.

The knowledge of new epigenetic mechanisms implicated in the development of epilepsy will provide a conceptual and mechanistic framework for future development of therapies tailored to prevent epilepsy (antiepileptogenic) or its progression (disease modifying).

## Author Contributions

AS put forward a hypothesis and wrote the manuscript; made analysis and interpretation of data; made a final approval of the version to be published; and agreed to be accountable for all aspects of the work. IS discussed hypothesis, wrote, and edited the manuscript; made substantial contributions to the conception of the work; made a final approval; and agreed to be accountable for all aspects of the work. MS discussed proposed hypothesis, wrote, and edited the manuscript; made analysis and interpretation of data; made a final approval of the version to be published; made a final approval; and agreed to be accountable for all aspects of the work. RS made contributions to the conception or design of the work; made a final approval of the version to be published; agreed to be accountable for all aspects of the work in ensuring questions related to the accuracy and integrity; and made interpretation of data. VS discussed proposed hypothesis, wrote, and edited the manuscript; made analysis and interpretation of data; made a final approval of the version to be published; made a final approval; and agreed to be accountable for all aspects of the work. RS made interpretation of data.

## Disclaimer

The views expressed in this article are those of the authors and do not necessarily reflect the position or policy of the Department of Veterans Affairs or the United States Government.

## Conflict of Interest Statement

The authors do not have any conflict of interest to declare regarding the contents of the article.
